# Bacterial ventriculoperitoneal shunt infections: changing trends in antimicrobial susceptibility, a 7-year retrospective study from Pakistan

**DOI:** 10.1186/s13756-023-01283-3

**Published:** 2023-08-08

**Authors:** Amina Akram Asif, Khalid Mahmood, Saba Riaz, Timothy McHugh, Sikander Sultan

**Affiliations:** 1 University of the Punjab, Lahore, Pakistan; 2 Lahore General Hospital, Punjab Institute of Neurosciences, Lahore, Pakistan; 3 University College London, London, UK

**Keywords:** Ventriculoperitoneal shunt Infections, Antimicrobial susceptibility, Stewardship program

## Abstract

**Background:**

Ventriculoperitoneal (VP) shunt infections in adults represent a severe complication and make treatment more challenging. Therefore, drug susceptibility patterns are crucial for therapeutic decisions and infection control in neurosurgical centers. This 7-year retrospective study aimed to identify the bacteria responsible for adult VP shunt infections and determine their drug susceptibility patterns.

**Methods:**

This single-center study was performed from 2015 to 2021 in Lahore, Pakistan, and included CSF cultures from VP shunt infections. Demographic data, causative organisms, and antimicrobial susceptibility testing results were collected. Multivariate analysis of variance (MANOVA) and two-sample t-tests were used to analyze and compare the antibiotic sensitivity trends over the study period.

**Results:**

14,473 isolates recovered from 13,937 CSF samples of VP shunt infections were identified and analyzed for their susceptibility patterns to antimicrobials. The proportion of Gram-negative and Gram-positive bacteria were 11,030 (76%) and 3443 (24)%, respectively. The predominant bacteria were *Acinetobacter* species (*n* = 5898, 41%), followed by *Pseudomonas* species (*n* = 2368, 16%) and coagulase-negative *Staphylococcus* (CoNS) (*n* = 1880, 13%). 100% of *Staphylococcus aureus (S.aureus)* and CoNS were sensitive to vancomycin and linezolid (*n* = 2580). However, 52% of *S. aureus* (719/1,343) were methicillin-resistant *Staphylococcus aureus* (MRSA). *Acinetobacter* showed maximum sensitivity to meropenem at 69% (2759/4768). *Pseudomonas* was 80% (1385/1863 sensitive to piperacillin-tazobactam, *Escherichia coli* (*E. coli)* showed 72% to amikacin (748/1055), while *Klebsiella* spp. was 57% (574/1170) sensitive to piperacillin-tazobactam. The sensitivity of piperacillin-tazobactam and meropenem for Gram-negative bacteria decreased significantly (*p* < 0.05) over 7 years, with 92.2% and 88.91% sensitive in 2015 and 66.7% and 62.8% sensitive in 2021, respectively.

**Conclusion:**

The significant decrease in the effectiveness of carbapenem and beta-lactam/beta-lactamase inhibitor combination drugs for the common Gram-negative causative agents of VP shunt infections suggests that alternative antibiotics such as colistin, fosfomycin, ceftazidime/avibactam, ceftolozane/tazobactam, and tigecycline should be considered and in consequence included in testing panels. Additionally, it is recommended to adopt care bundles for the prevention of VP shunt infection.

## Introduction

Ventriculoperitoneal (VP) shunt insertion is one of the most frequently performed neurosurgical interventions worldwide [1, 2], where shunt insertion can restore the elevated intracranial pressure associated with hydrocephalus and is chiefly used for its management [3, 4]. Unfortunately, complications related to the VP shunt placement are common. One severe consequence is the development of infection after shunting, despite the availability of new antibiotics and advanced neurosurgical techniques. Shunt infection rates range from 5 to 15% of the patients undergoing the procedure and are often associated with adverse outcomes [5]. Independent risk factors for VP shunt infections include the initial indication of shunt placement, revision or replacement for dysfunction, previous shunt-associated infection, postoperative CSF leakage, extreme age groups, procedure duration, the neurosurgeon's experience, and use of a neuro endoscope [6–8] More than 60% of the shunt infections occur within the first four to five weeks after the shunt placement. However, late shunt infections after some years are also observed [1, 2, 7]. Early shunt infections are often initiated during shunt insertion whereas, late infections are associated with unconnected pathologies, e.g. peritonitis and bowel perforation [7, 9]. The VP shunt infection frequently leads to ventriculitis and meningitis [10]. Any delay in effective treatment can have a poor prognosis with a mortality rate of 20–50% [10, 11]. Therefore, when the infection is clinically apparent, antimicrobial therapy should be started immediately along with the removal of shunt where applicable [12, 13]. and empirical antimicrobial treatment based on regional epidemiology, the prevalence of potential bacteria, and antimicrobial susceptibility patterns is essential [14, 15]. During the last decade, the infectious bacterial spectrum in VP shunt infection has started shifting from previously common causative agents such as *Staphylococcus aureus*, coagulase-negative *Staphylococcus*, and *Enterococcus* Gram-positive bacteria, to Gram-negative bacilli, especially *Acinetobacter* species, *Pseudomonas* species, and Enterobacterales [10].

Prescribing empirical antibiotics for this acute illness remains challenging. Antimicrobial resistance (AMR) surveillance data of microorganisms and the antibiotic susceptibility profile should be made available as a limited number of drugs can penetrate the central nervous system (blood–brain barrier). The emergence of multi-drug resistance (MDR), extensively drug-resistant (XDR), and even pan-drug-resistant microorganisms is catastrophic [11]. The mortality rate can extend to 60% to 70% in neurosurgical infection with carbapenem-resistant Gram-negative bacteria [10, 11]. Once the causative pathogen and antimicrobial susceptibility pattern have been determined by microbiology, every effort should be made to tailor the empiric treatment as per the sensitivity spectrum for the particular bacterium [16].

Our literature review revealed that limited international studies had been conducted on antibiotic susceptibility of VP shunt isolates in adults [5, 17], without any previous investigation in Pakistan, the fifth most populous country in the world. Herein, to fill the knowledge gap, we report epidemiological surveillance data at the leading neurosurgical institute of the country and assess the causative pathogens and their antimicrobial susceptibility patterns of antibiotics for VP shunt infections.

## Materials and methods

### Setting

The Punjab Institute of Neurosciences (PINS), Lahore General Hospital,located in the Lahore city of Punjab Province, is the largest and premier specialized neurosurgical center in Pakistan for more than fifty years. It has a capacity of five hundred beds and equipped with eight theatres for elective and two theatres for emergency surgeries. Each day, 700–800 outpatients and emergency patients are taken care of with output of nearly 7000 elective brain and spine operations in a year. Out of these, 10 to 20% of patients have shunt-related illnesses and they come in to PINS either directly or are referred to as complicated cases from other healthcare centers, specifically from Punjab province (population around 110 million) and generally from all around Pakistan (population around 207.8 million). [18]

### Study design and data collection

**Fig. 1:**
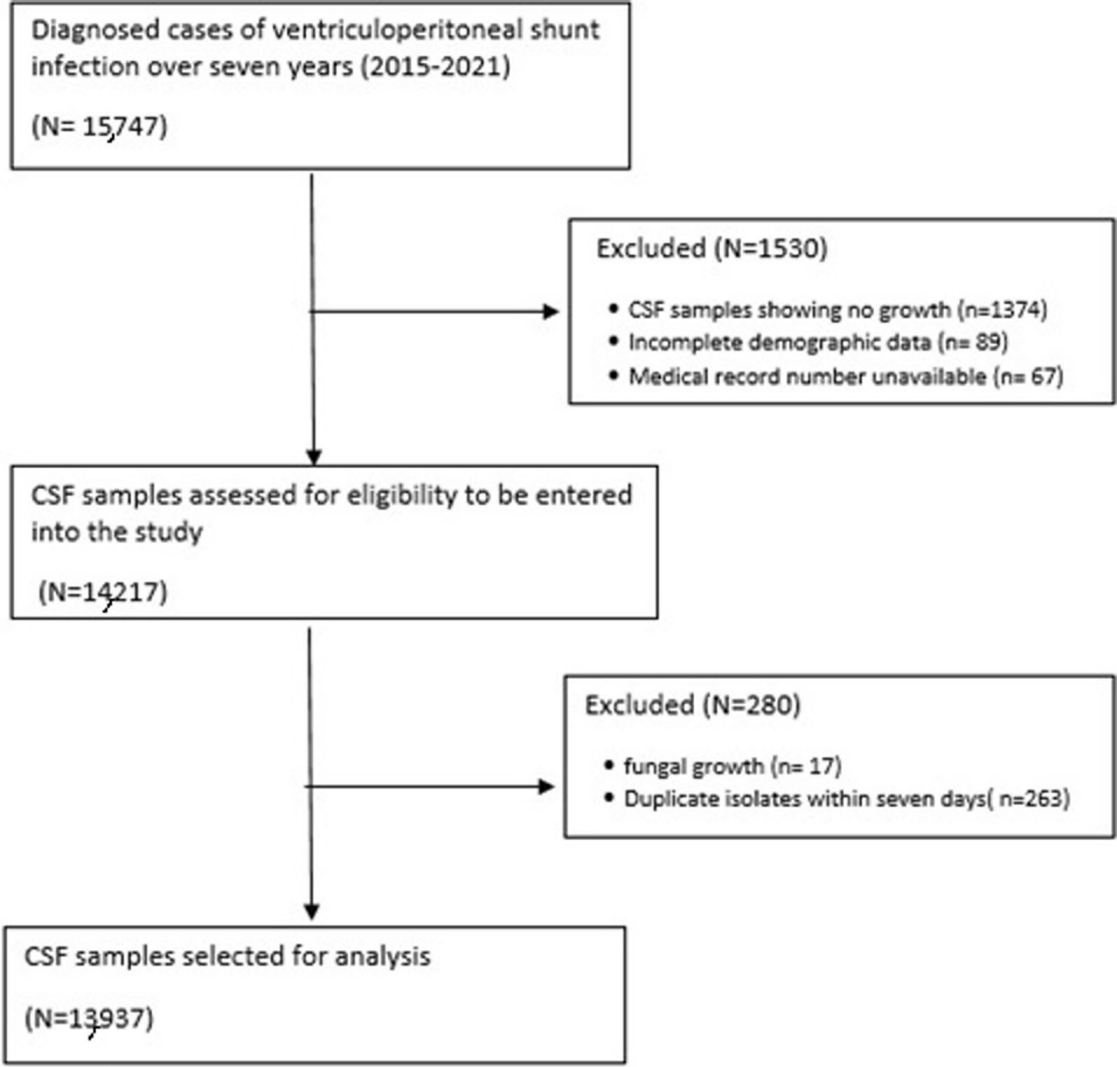
Flow chart for cerebrospinal fluid (CSF) sample selection to be included in the study

In this retrospective study, we reviewed the records of clinically diagnosed cases of VP shunt infection and their respective reports of CSF culture and sensitivity from January 2015 to December 2021. The VP shunts inserted were plain. Some samples were excluded from the study based on incomplete information, for example, duplicate isolates within 7 days, and mismatched medical record numbers (Fig. 1). CSF samples from patients with diagnosed bacterial VP shunt infection and complete demographic and medical information were included in the study for final evaluation. Extracted data showed: age, gender, organism identified, and antimicrobial susceptibility patterns.

Antimicrobial susceptibility results were further analyzed only for the isolates recovered from the CSF of patients with VP shunt infection, each year from 2015 to 2021 (bacteria 30 or more) [19]. So data included for Gram-negative bacteria, including *Acinetobacter* species, *Pseudomonas* species, *Escherichia coli, Klebsiella* species, and Gram-positive bacteria (*Staphylococcus aureus,* and Coagulase-negative *Staphylococcus*).

### Identification of bacterial isolates and antimicrobial susceptibility testing

All CSF samples were processed in the microbiology laboratory according to the standard operating procedure [20]. Briefly, CSF samples were inoculated on sheep blood agar, chocolate agar, and MacConkey agar. Bacterial identification was performed by analytical profile index (API) (Biomerieux) [20]. Antibiotic susceptibility was determined by the Kirby-Bauer disc diffusion method and minimum inhibitory concentration (MIC) determination according to the Clinical and Laboratory Standards Institute (CLSI) guidelines [13] The laboratory deployed antibacterial testing of the drugs per the CLSI criteria for each bacterium and the laboratory's availability of antibiotic discs for the given years. Agents administered by oral routes only, first and second-generation cephalosporins and cephamycins, doripenem, ertapenem, imipenem and lefamulin, clindamycin, macrolides, tetracyclines, fluoroquinolones were excluded for the CSF isolates as per CLSI recommendation [13]. Based on a review of clinical practice in PINS throughout the study, the following antibiotics were tested, amikacin, gentamicin, cotrimoxazole (trimethoprim-sulphamethoxazole), ceftriaxone, ceftazidime, cefoperazone, cefotaxime, cefepime, piperacillin, ampicillin, amoxicillin-clavulanic acid, piperacillin-tazobactam, ampicillin-sulbactam, meropenem, oxacillin, penicillin, vancomycin, and linezolid.

### Statistical analysis

The statistical results for continuous variables were presented as mean ± SD, range, or median (IQR) according to the statistical distribution. Categorical variables were presented as frequencies and percentages. Antimicrobial Susceptibility patterns of the bacteria were presented over time (years). The difference in sensitivity trends between 2015 and 2021 was examined using the multivariate analysis of variance (MANOVA), and two-sided *p*-values < 0.05 were considered statistically significant. The percentage of sensitive isolates was calculated as the sum of all sensitive bacteria (excluding both intermediately susceptible and resistant isolates) relative to the total number of bacteria tested against a particular drug. The sensitivity percentage was compared between 2015 and 2021 by a two-sample *t*-test and *p*-values < 0.05 were considered statistically significant. SPSS (IBM SPSS Statistics 23.0), Minitab version 17, and Microsoft Excel 2019 were used for statistical analyses and graphical presentation.

## Results

**Fig. 2:**
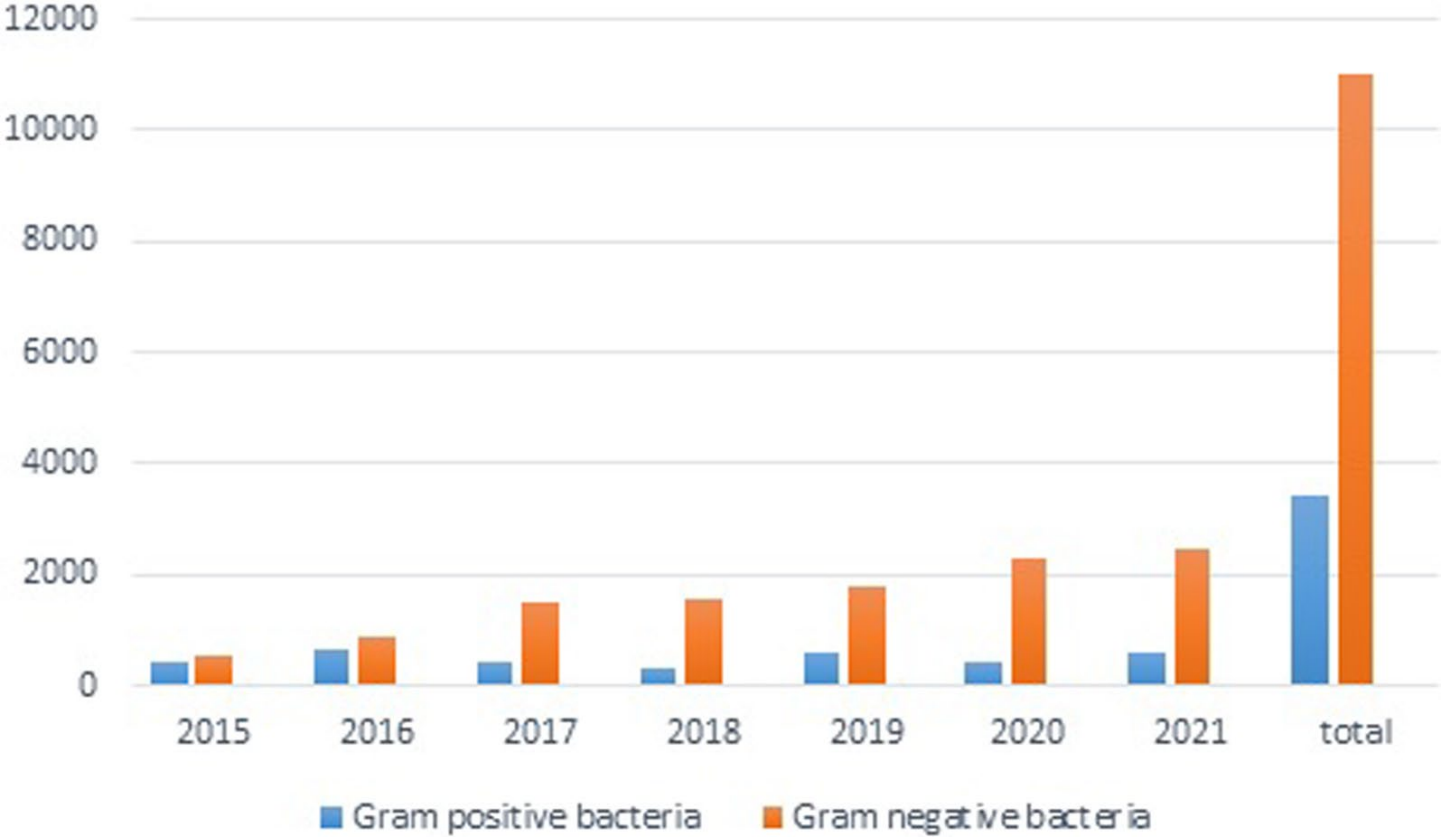
Gram-negative bacteria rate progression as compared to Gram-positive bacteria in VP shunt infections over 7 years (2015–2021) (N = 14,473)

**Fig. 3:**
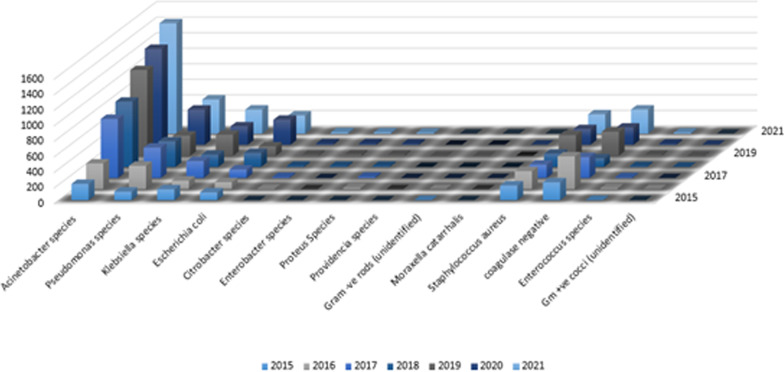
Frequency of isolated bacteria causing VP shunt infections in adults over 7 years (2015–2021) (N = 14,473)

During 7 years (2015–2021), 14,473 aerobic bacterial isolates were recovered from 13,937 CSF samples from patients with clinically diagnosed VP shunt infection; 536 (3.7%) of the CSF specimens showed the growth of more than one organism. The CSF samples came from 8,514 (59.9%) males and 5959 (40.1%) females with a mean age of 36.7 ± 19.3 years (range 15–92 years). Of 14,473 bacterial isolates analyzed, 11,030 (76%) were Gram-negative bacteria, and 3,443 (24%) were Gram-positive. The proportion of Gram-negative bacteria relative to the total number of bacteria increased over the course of the study: 57.2%, 77%, and 85.3 in 2015, 2017, and 2021 respectively (Fig. 2). *Acinetobacter* species were found to be predominant (41%) from 2015 to 2021, followed by *Pseudomonas* species (16%), Coagulase-negative *Staphylococcus* (13%), *Staphylococcus aureus* (10%), *Klebsiella* species (10%), *Escherichia coli* (8%) and others. An increasing trend was observed in the *Acinetobacter* species (Fig. 3).

### Trends of antimicrobial susceptibility among bacteria

A total of 14,473 bacteria were tested against 14 clinically significant antimicrobials. Bacteria showed an overall susceptibility of ≥ 48.1%, with Gram-positive being 57.1% sensitive and Gram-negative bacteria being 39.0% sensitive. Antimicrobial susceptibility patterns for each bacterial species are presented in (Table 1). In 7 years, the highest frequency of sensitivity of Gram-negative pathogens to antibiotics was seen towards meropenem, piperacillin-tazobactam, and ampicillin-sulbactam by *Acinetobacter*, 69%, 64%, and 53%, respectively; piperacillin-tazobactam, meropenem, and amikacin by *Pseudomonas*, 80%, 71%, and 67% respectively; piperacillin-tazobactam, amikacin, and meropenem by *Klebsiella*, 57%,56%, and 50% respectively; amikacin, meropenem, and piperacillin-tazobactam by *E. coli*, 72%, 68%, and 67% respectively. The Gram-positive bacteria, including *S. aureus* and CoNS, were seen to be completely sensitive (100%) toward vancomycin and linezolid. 52% of *S. aureus* were MRSA, while methicillin resistance was found in 69.5% of CoNS.

**Table 1:** Antimicrobial susceptibility rates among the bacteria causing ventriculoperitoneal shunt infections over 7 years (2015–2021)

*Acinetobacter* species	2015			2016			2017			2018			2019			2020			2021			Total			*p*-value*
	*N* = 211			*N* = 330			*N* = 762			*N* = 840			*N* = 1100			*N* = 1230			*N* = 1425			*N* = 5898			
	*T*	*S*	*S* %	*T*	*S*	*S* %	*T*	*S*	*S*%	*T*	*S*	*S*%	*T*	*S*	*S*%	*T*	*S*	*S* %	*T*	*S*	*S* %	*T*	*S*	S %	
Amikacin	101	52	51.5	271	130	48.0	636	322	50.6	840	378	45.0	1082	510	47.1	1220	490	40.2	1410	503	35.7	5560	2385	45.4	0.965
Cefepime	211	73	34.6	271	52	19.2	636	134	21.1	820	161	19.6	1100	145	13.2	1220	140	40.2	1410	211	15.0	5668	916	23.3	0.553
Cefotaxime	90	21	23.3	157	9	5.7	231	23	10.0	193	12	6.2	660	54	8.2	1160	80	40.2	1425	160	11.2	3916	359	15.0	0.925
Ceftazidime	149	13	8.7	300	13	4.3	762	110	14.4	810	101	12.5	1100	113	10.3	1230	110	40.2	1425	177	12.4	5776	637	14.7	0.689
Ceftriaxone	211	15	7.1	300	20	6.7	379	31	8.2	793	72	9.1	1073	127	11.8	1220	130	40.2	1425	155	10.9	5401	550	13.4	0.295
Cotrimoxazole	211	41	19.4	330	82	24.9	120	10	8.3	810	133	16.4	904	194	21.5	1233	220	40.2	1410	332	23.6	5018	1012	22.0	0.867
Gentamicin	100	31	31.0	65	23	35.4	181	63	34.8	840	251	29.9	1100	344	31.3	1230	450	40.2	1425	491	34.5	4941	1653	33.9	0.873
Meropenem	20	20	100.0	70	61	87.1	231	151	65.4	840	592	70.5	1100	596	54.2	1220	673	40.2	1287	666	51.8	4768	2759	67.0	0.576
Piperacillin_tazobactam	180	157	87.2	254	182	71.7	334	264	79.0	838	597	71.2	1100	571	51.9	1220	570	40.2	1410	603	42.8	5336	2944	63.4	0.776
Ampicillin_sulbactam	211	140	66.4	271	211	77.9	340	280	82.4	780	287	36.8	1100	532	48.4	1230	410	40.2	1425	330	23.1	5357	2190	53.6	0.845

*Pseudomonas* species	2015			2016			2017			2018			2019			2020			2021			Total			*p*-value*
	*N* = 110			*N* = 302			*N* = 393			*N* = 330			*N* = 340			*N* = 453			*N* = 440			*N* = 2368			
	*T*	*S*	*S*%	*T*	*S*	*S*%	*T*	*S*	*S*%	*T*	*S*	*S*%	*T*	*S*	*S*%	*T*	*S*	*S*%	*T*	*S*	*S*%	*T*	*S*	*S*%	
Amikacin	72	51	70.8	284	188	66.2	393	288	73.3	327	222	67.9	340	261	76.8	453	311	68.7	440	211	48.0	2309	1532	67.4	0.742
Cefepime	100	63	63.0	221	89	40.3	313	170	54.3	327	181	55.4	340	209	61.5	450	161	35.8	434	142	32.7	2185	1015	49.0	0.818
Ceftazidime	83	21	25.3	289	90	31.1	360	99	27.5	325	129	39.7	340	121	35.6	450	101	22.4	440	127	28.9	2287	688	30.1	0.984
Gentamicin	37	11	29.7	37	14	37.8	360	101	28.1	330	158	47.9	340	175	51.5	453	258	57.0	440	178	40.5	1997	895	41.8	0.807
Meropenem	110	87	79.1	71	60	84.5	128	93	72.7	310	182	58.7	60	41	68.3	450	267	59.3	62	45	72.6	1191	775	70.7	0.852
Piperacillinn	110	81	73.6	302	124	41.1	56	21	37.5	220	111	50.5	332	185	55.7	450	172	38.2	440	150	34.1	1910	844	47.2	0.926
Piperacillin_tazobactam	100	89	89.0	160	155	96.9	56	51	91.1	330	243	73.6	340	261	76.8	450	341	75.8	427	245	57.4	1863	1385	80.1	0.991

*Klebsiella* species	2015			2016			2017			2018			2019			2020			2021			Total			*p*-value*
	*N* = 141			*N* = 109			*N* = 211			*N* = 153			*N* = 271			*N* = 240			*N* = 310			*N* = 1435			
	*T*	*S*	*S*%	*T*	*S*	*S*%	*T*	*S*	*S*%	*T*	*S*	*S*%	*T*	*S*	*S*%	*T*	*S*	*S*%	*T*	*S*	*S*%	*T*	*S*	*S*%	
Amikacin	92	71	77.2	72	39	54.2	211	142	67.3	153	73	47.7	271	129	47.6	240	110	45.8	308	163	52.9	1347	727	56.1	0.529
Amoxicillin_clavulanicacid	140	32	22.9	109	22	20.2	190	10	5.3	153	12	7.8	269	20	7.4	240	21	8.8	308	31	10.1	1409	148	11.8	0.833
Cefepime	141	19	13.5	68	10	14.7	180	22	12.2	153	21	13.7	260	60	23.1	233	44	18.9	308	69	22.4	1343	245	16.9	0.534
Cefoperazone	140	39	27.9	109	33	30.3	211	53	25.1	153	39	25.5	271	91	33.6	230	51	22.2	310	31	10.0	1424	337	24.9	0.645
Ceftazidime	127	64	50.4	100	31	31.0	180	52	28.9	153	31	20.3	270	21	7.8	240	23	9.6	310	48	15.5	1380	270	23.3	0.588
Ceftriaxone	129	52	40.3	109	9	8.3	180	19	10.6	150	21	14.0	270	20	7.4	240	21	8.8	310	29	9.4	1388	171	14.1	0.726
Cotrimoxazole	110	41	37.3	109	41	37.6	38	11	29.0	153	19	12.4	268	29	10.8	240	47	19.6	306	31	10.1	1224	219	22.4	0.413
Gentamicin	20	10	50.0	51	23	45.1	180	65	36.1	147	55	37.4	270	122	45.2	240	89	37.1	307	100	32.6	1215	464	40.5	0.506
Meropenem	22	16	72.7	109	50	45.9	157	79	50.3	153	66	43.1	271	110	40.6	240	101	42.1	300	142	47.3	1252	564	48.9	0.61
Piperacillin_tazobactam	91	70	76.9	100	79	79.0	19	13	68.4	153	67	43.8	270	119	44.1	227	92	40.5	310	134	43.2	1170	574	56.6	0.657

*Escherichia coli*	2015			2016			2017			2018			2019			2020			2021			Total			*p*-value*
	*N* = 103			*N* = 92			*N* = 103			*N* = 177			*N* = 127			*N* = 320			*N* = 232			*N* = 1154			
	*T*	*S*	*S*%	*T*	*S*	*S*%	*T*	*S*	*S*%	*T*	*S*	*S*%	*T*	*S*	*S*%	*T*	*S*	*S*%	*T*	*S*	*S*%	*T*	*S*	*S*%	
Amikacin	57	50	87.7	71	59	83.1	76	38	50.0	177	120	67.8	127	83	65.4	320	201	62.8	227	197	86.8	1055	748	71.9	0.897
Amoxicillin_clavulanicacid	103	11	10.7	92	11	12.0	71	10	14.1	170	20	11.8	120	20	16.7	320	25	7.8	232	30	12.9	1108	127	12.3	0.706
Ampicillin	29	9	31.0	92	10	10.9	110	11	10.0	80	10	12.5	110	11	10.0	320	32	10.0	232	28	12.1	973	111	13.8	0.939
Cefepime	90	31	34.4	50	10	20.0	77	14	18.2	177	80	45.2	127	22	17.3	320	57	17.8	232	39	16.8	1073	253	24.3	0.564
Cefoperazone	40	11	27.5	50	13	26.0	40	10	25.0	177	100	56.5	120	50	41.7	320	41	12.8	232	81	34.9	979	306	32.1	0.584
Ceftazidime	67	29	43.3	92	28	30.4	103	11	10.7	177	70	39.6	120	27	22.5	247	32	13.0	232	27	11.6	1038	224	24.4	0.876
Ceftriaxone	103	33	32.0	90	11	12.2	77	9	11.7	175	40	22.9	120	11	9.2	320	30	9.4	227	33	14.5	1112	167	16.0	0.613
Cotrimoxazole	37	9	24.3	50	13	26.0	40	10	25.0	177	50	28.3	120	20	16.7	320	61	19.1	232	31	13.4	976	194	21.8	0.656
Gentamicin	80	70	87.5	39	27	69.2	40	20	50.0	177	100	56.5	127	53	41.7	320	189	59.1	232	140	60.3	1015	599	60.6	0.362
Meropenemn	50	41	82.0	39	33	84.6	55	37	67.3	134	97	72.4	120	70	58.3	320	177	55.3	220	127	57.7	938	582	68.2	0.406
Piperacillin_tazobactam	77	63	81.8	50	39	78.0	40	29	72.5	134	103	76.9	120	57	47.5	320	163	50.9	232	139	59.9	973	593	66.8	0.892

*Staphylococcus aureus*	2015			2016			2017			2018			2019			2020			2021			Total			*p*-value*
	*N* = 179			*N* = 231			*N* = 171			*N* = 176			*N* = 270			*N* = 203			*N* = 257			*N* = 1487			
	*T*	*S*	*S*%	*T*	*S*	*S*%	*T*	*S*	*S*%	*T*	*S*	*S*%	*T*	*S*	*S*%	*T*	*S*	*S*%	*T*	*S*	*S*%	*T*	*S*	*S*%	
Amikacin	44	22	50.0	210	155	73.8	170	109	64.1	33	18	54.6	34	18	52.9	110	40	36.4	250	104	41.6	851	466	53.3	0.432
Oxacillin **	179	107	59.8	231	137	59.3	170	90	52.9	162	79	48.8	267	94	35.2	97	50	51.6	237	67	28.3	1343	624	48.0	0.765
Cotrimoxazole	140	27	19.3	210	50	23.8	170	52	30.6	170	50	29.4	270	110	40.7	200	81	40.5	240	116	48.3	1400	486	33.2	0.706
Gentamicin	140	50	35.7	27	11	40.7	170	79	46.5	160	83	51.9	270	109	40.4	200	132	66.0	250	87	34.8	1217	551	45.1	0.526
Penicillin	179	27	15.1	210	58	27.6	170	41	24.1	160	72	45.0	270	69	25.6	167	33	19.8	238	45	18.9	1394	345	25.2	0.827
Linezolid	193	193	100.0	231	231	100.0	137	137	100.0	176	176	100.0	270	270	100.0	203	203	100.0	257	257	100.0	1467	1467	100.0	0.989
Vancomycin	87	87	100.0	210	210	100.0	171	171	100.0	162	162	100.0	270	270	100.0	167	167	100.0	257	257	100.0	1324	1324	100.0	0.989

Coagulase-negative *Staphylococcus*	2015			2016			2017			2018			2019			2020			2021			Total			*p*-value*
	*N* = 230			*N* = 422			*N* = 270			*N* = 117			*N* = 310			*N* = 220			*N* = 311			*N* = 1880			
	*T*	*S*	*S*%	*T*	*S*	*S*%	*T*	*S*	*S*%	*T*	*S*	*S*%	*T*	*S*	*S*%	*T*	*S*	*S*%	*T*	*S*	*S*%	*T*	*S*	*S*%	
Amikacin	230	159	69.1	412	287	69.7	267	158	59.2	117	83	70.9	78	47	60.3	158	71	44.9	297	113	38.1	1559	918	58.9	0.761
Oxacillin ***	187	64	34.2	422	120	28.4	267	90	33.7	117	45	38.5	310	125	40.3	120	30	25.0	311	41	13.2	1734	515	30.5	0.601
Cotrimoxazole	187	111	59.4	387	179	46.3	270	110	40.7	110	40	36.4	310	121	39.0	220	83	37.7	311	98	31.5	1795	742	41.6	0.964
Gentamicin	84	41	48.8	420	210	50.0	270	140	51.9	110	41	37.3	300	139	46.3	220	83	37.7	300	147	49.0	1704	801	45.9	0.794
Linezolid	212	212	100.0	420	420	100.0	178	178	100.0	110	110	100.0	310	310	100.0	220	220	100.0	300	300	100.0	1750	1750	100.0	0.989
Penicillin	230	27	11.7	422	69	16.4	267	35	13.1	103	17	16.5	310	87	28.1	90	17	18.9	311	37	11.9	1733	289	16.7	0.315
Vancomycin	56	56	100.0	120	120	100.0	123	123	100.0	97	97	100.0	300	300	100.0	120	120	100.0	297	297	100.0	1113	1113	100.0	0.989

*N* Number of bacteria causing VP shunt infections, *T* Number of tested isolates, *S* Number of susceptible isolates, S(%) percentage of susceptible isolates

*Multivariate analysis of variance (MANOVA) for non-susceptible trend

**Isolates resistant to oxacillin (interpreted with cefoxitin disc diffusion) are defined as MRSA (Methicillin-resistant *Staphylococcus aureus*). They are considered resistant to other beta-lactam agents, i.e., penicillins, beta-lactam combination agents, cephems (with the exception of ceftaroline), and carbapenems [15].

***Isolates resistant to oxacillin (interpreted with cefoxitin disc diffusion) are methicillin-resistant. They are considered resistant to other beta-lactam agents, i.e., penicillins, beta-lactam combination agents, cephems (with the exception of ceftaroline), and carbapenems [15].

Conversely, the lowest frequency of sensitivity of Gram-negative bacteria to antimicrobials was seen towards amoxicillin-clavulanic acid by *Klebsiella* species and *E. coli*, being 11.8% and 12.3% sensitive, respectively: ceftriaxone by *Acinetobacter* species (13.4%) and Ceftazidime by *Pseudomonas* species (30.1%). Cumulatively, the frequency of sensitivity of all Gram-negative bacteria was less than 30% towards the third-generation cephalosporins (ceftazidime, cefotaxime, ceftriaxone, cefoperazone). However, in Gram-positive bacteria, both *S. aureus* and CoNS were least sensitive towards penicillin, with 25.2% and 16.7% sensitivity, respectively.

### Trends of antibiotics

**Fig. 4:**
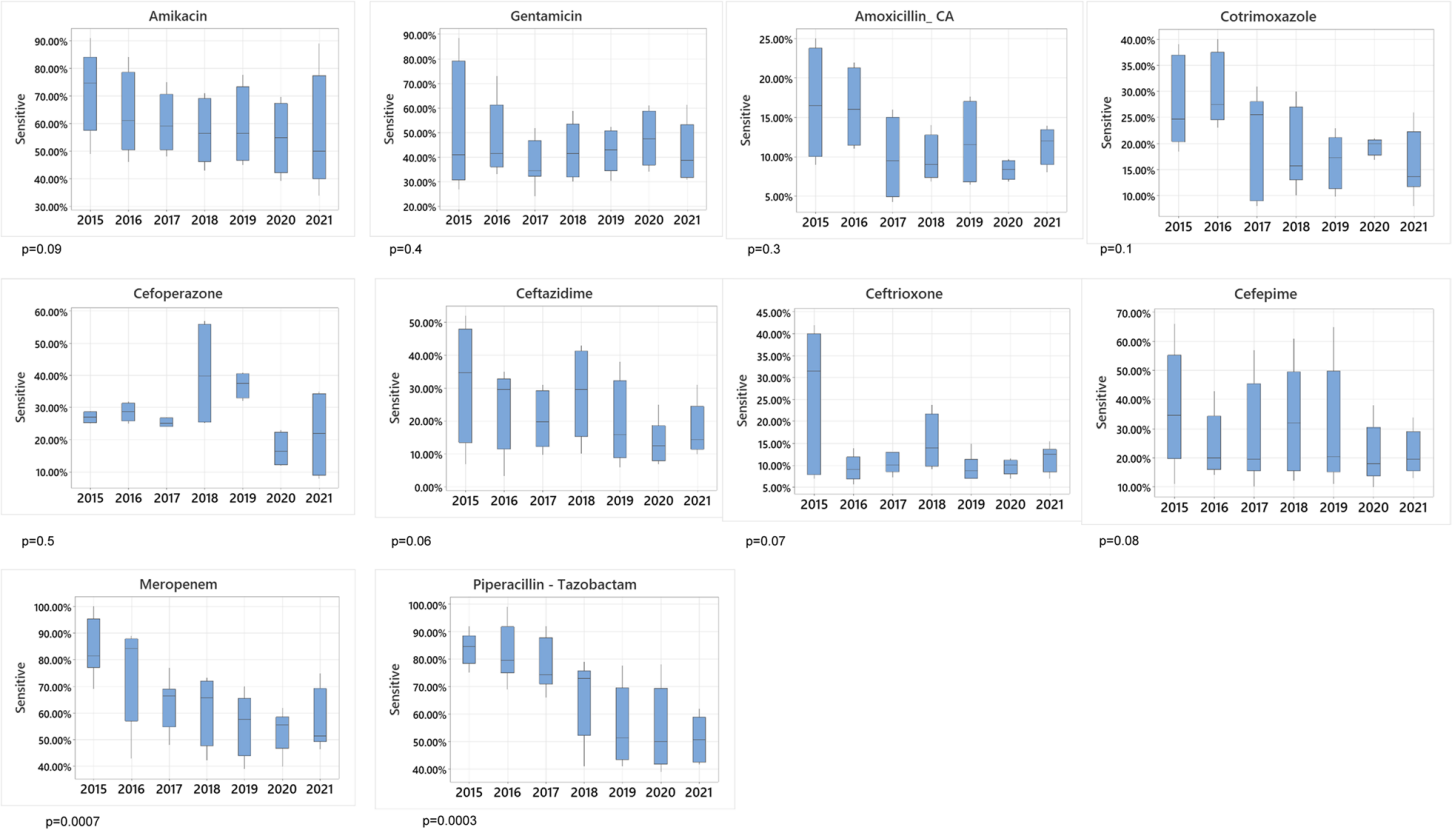
Boxplots showing yearly antimicrobial effectiveness of antibiotics in terms of sensitivity for Gram-negative bacteria cumulatively (for which the drug has been reported) each year. For each antibiotic, boxes represent the sensitivity rate at the 25–75th percentiles (interquartile range), and the ends of vertical lines represent values at the 10^–^90th percentiles for the respective year. Horizontal lines represent median values for each year. The comparison of the efficacy of the drug between 2015 and 2021 was done by a two-sample *t*-test. *P* =  < 0.05 was considered significant

Year-wise frequency of sensitivity of the drugs commonly prescribed during the study period for Gram-negative bacteria against which the drugs have been reported during the study period (Fig. 4) showed a falling trend of sensitivity over 7 years (2015–2021). A significant decrease in the frequency of sensitivity for piperacillin-tazobactam (*p* = 0.0003) and meropenem (*p* = 0.0007) by all the Gram-negative bacteria collectively occurred in 2021 compared to 2015, piperacillin-tazobactam losing its sensitivity by 32.92% and meropenem by 26.11%. A prominent insignificant decrease in sensitivity frequency was shown by amikacin, 15.97%, followed by third-generation and fourth-generation cephalosporins, losing 14.90%, 14.82%, and 14.66% sensitivity by ceftriaxone, ceftazidime, and cefepime respectively. Cotrimoxazole showed 11.33% less sensitivity in 2021 compared to 2015, while gentamicin lost its efficacy by 7.06%. Amoxicillin-clavulanic acid showed low sensitivity throughout the 7 years without prominent variation, being 16.77% sensitive in 2015 and 11.50% in 2021, with a 5.25% loss in susceptibility.

## Discussion

Infection is a severe complication after VP shunting which may lead to prolonged hospital stay, increased medical costs, or even death. [2]. However, the data regarding the etiology of this infection is scarce, especially in the adult population. In this study, CSF culture results and antibiotic susceptibility were analyzed over 7 years. Here, 3.7% of the CSF samples revealed more than one organism, similar to some previous studies [21, 22], although, a single organism has been reported in the literature more often [23–25]. This discrepancy may be due to reporting in clinical practice where more than one organism is often considered a contaminated sample and reported as such. The changing spectrum of VP shunt infection-causing bacteria from Gram-positive to Gram-negative, as seen in our study as well (Fig. 2), might be because of the complex neurosurgery, neurocritical care, extended hospital stays, healthcare-associated infections, and antibiotic prophylaxis targeting Gram-positive bacteria [26–29].

In this study, most of the Gram-negative pathogens, including *Acinetobacter* spp.*.*, *Pseudomonas* spp, *Klebsiella* spp, and *Escherichia coli*, showed an overall trend of increased resistance towards all the drugs used for the empirical treatment of VP shunt infection included in this study. Meropenem is the primary empirical and targeted treatment, consistent with the recommended guidelines [5]. Other recommended antimicrobial agents [5] include cefepime and ceftazidime. However, PINS rarely uses them as empirical treatments because of their high resistance rates. (Table 1). Unfortunately, the most significant decrease in sensitivity was seen for Gram-negative bacilli collectively (*p* < 0.05) against meropenem (26.11%) and piperacillin-tazobactam (32.92%). When individual isolates were tested for meropenem susceptibility, *Acinetobacter* susceptibility was reduced by 50% over the course of the study. Sensitivity to meropenem declined for *Klebsiella* spp and *E. coli* by 25.4% and 24.27%, respectively. High-level carbapenem resistance is on the rise and has been reported in the literature [10, 30, 31]. Of all the antibiotics compared for the difference in susceptibility over the study period, gentamicin showed the least change, being 50% sensitive in 2015 and 42% sensitive in 2021. Although such a phenomenon in treating VP shunt infections has not been reported before, further studies should be done to assess its significance.

We had some limitations while concluding the results. As it is a retrospective study and our center receives referral infected and complicated cases from other healthcare facilities as well, we donot have exact data about how many VP shunt infections were relapses or reinfections.

Based on our results, the management of patients with VP shunt infections should be guided by some fundamental principles for improving empirical therapy. The currently prescribed drug (meropenem) gives Gram-negative coverage, but it has lost its efficacy considerably. Therefore, antibiotics including colistin, fosfomycin, ceftazidime/avibactam, ceftolozane/tazobactam, and tigecycline should be evaluated to have more effective treatment of infections caused by multidrug-resistant Gram-negative bacilli. However, the intravenous (IV) administration of antibiotics like colistin and tigecycline is associated with a very low CNS transfer. Consequently, a concomitant intrathecal or intraventricular administration route is required for the treatment of severe ventriculitis in patients with VP shunt infection [16]. It should be noted that although tigecycline and colistin have been used clinically for the last two years in our center for highly drug-resistant Gram-negative bacteria in VP shunt infections, data about their susceptibility patterns are unavailable due to inadequate guidelines on reporting these drugs. The synergistic action of antibiotics like meropenem–amikacin and meropenem–colistin combinations, ampicillin-sulbactam, and aminoglycosides combination therapy, should be explored. Furthermore, the clinical literature is emerging on using extended-infusion β-lactams to treat Gram-negative bacteria, especially with cefepime, piperacillin-tazobactam, and carbapenems (meropenem, imipenem, and doripenem). One of the key advantages of extended-infusion β-lactams is the ability to achieve drug concentrations above the MIC for a longer time for less susceptible organisms, especially those with a MIC between 4 and 16 µg/mL [32]. In addition, according to Infectious Diseases Society of America (IDSA) practice guidelines [33], intrathecal administration of anti-infectives should be considered for patients with healthcare-associated ventriculitis and meningitis in which the infection responds poorly to systemic antimicrobial therapy alone despite shunt removal in the setting of highly resistant organisms susceptible only to antibiotics with poor CSF penetration or in situations where devices cannot be removed.

In addition to addressing infections, we suggest the implementation of care bundles to decrease the frequency of VP shunt infections. Interventions that combine different prevention strategies appeared to be effective in certain settings. These bundles should include the enforcement of strict infection control protocols, emphasizing proper hand washing techniques while scrubbing and the use of strict sterile techniques during surgery, among other measures. We advocate for the use of antibiotic-impregnated shunt devices as they have the potential to reduce the incidence of CSF shunt infections [5, 34]. Furthermore, we support hair clipping instead of shaving, minimal trafficking during surgery, double gloving by all team members, the use of antibiotic-impregnated sutures and considering injecting vancomycin/gentamicin into the shunt reservoir as these measures have been shown to be effective in reducing the incidence of CSF infections [35].

## Data Availability

All data generated or analyzed during this study are included in this published article or are available from the corresponding author on request.
